# European aid and health system strengthening: an analysis of donor approaches in the DRC, Ethiopia, Uganda, Mozambique and the global fund

**DOI:** 10.1080/16549716.2019.1614371

**Published:** 2019-05-28

**Authors:** Lies Steurs

**Affiliations:** Centre for EU Studies, Department of Political Sciences, Ghent University, Gent, Belgium

**Keywords:** European Union, European donors, health system strengthening, global health, development cooperation, international health assistance, global fund

## Abstract

**Background**: In the field of international health assistance (IHA), there is a growing consensus on the limits of disease-specific interventions and the need for more health system strengthening (HSS). European donors are considered to be strong supporters of HSS. Nevertheless, little is known about how their support for HSS translates into concrete policies at partner country level. Furthermore, as development cooperation is a shared policy between the EU and its Member States, it remains unclear to what extent European donors share a similar approach.

**Objective**: This article reviews a PhD thesis on European aid and HSS. The thesis investigated (1) the approaches of European donors towards IHA, and (2) the extent to which there are similarities or differences between them. An original analytical framework was developed to make a fine-grained analysis of European donors’ approaches in the DRC, Ethiopia, Uganda and Mozambique. In addition, the relation of European donors with the Global Fund was investigated.

**Methods**: An abductive research approach was used during which literature review, data generation, analysis and research design mutually influenced each other. The research built on a wide range of empirical data, including semi-structured interviews with 123 respondents, policy documents and descriptive statistical analysis.

**Results and conclusion**: Four ‘types’ of European donors were identified, which vary in their focus (issue-specific versus comprehensive) and their level of support to and involvement of recipient states. Despite this heterogeneity at a specific level, there is still a general degree of ‘unity’ among European donors, especially compared with the US. Yet, there are signs that the ‘transatlantic’ divide on HSS may be converging, as European donors tend to focus more explicitly on result-oriented approaches traditionally associated with the US and Global Health Initiatives. Consequently, European donors play a limited role in bringing HSS more to the forefront in IHA.

## Background

**Figure 1.**
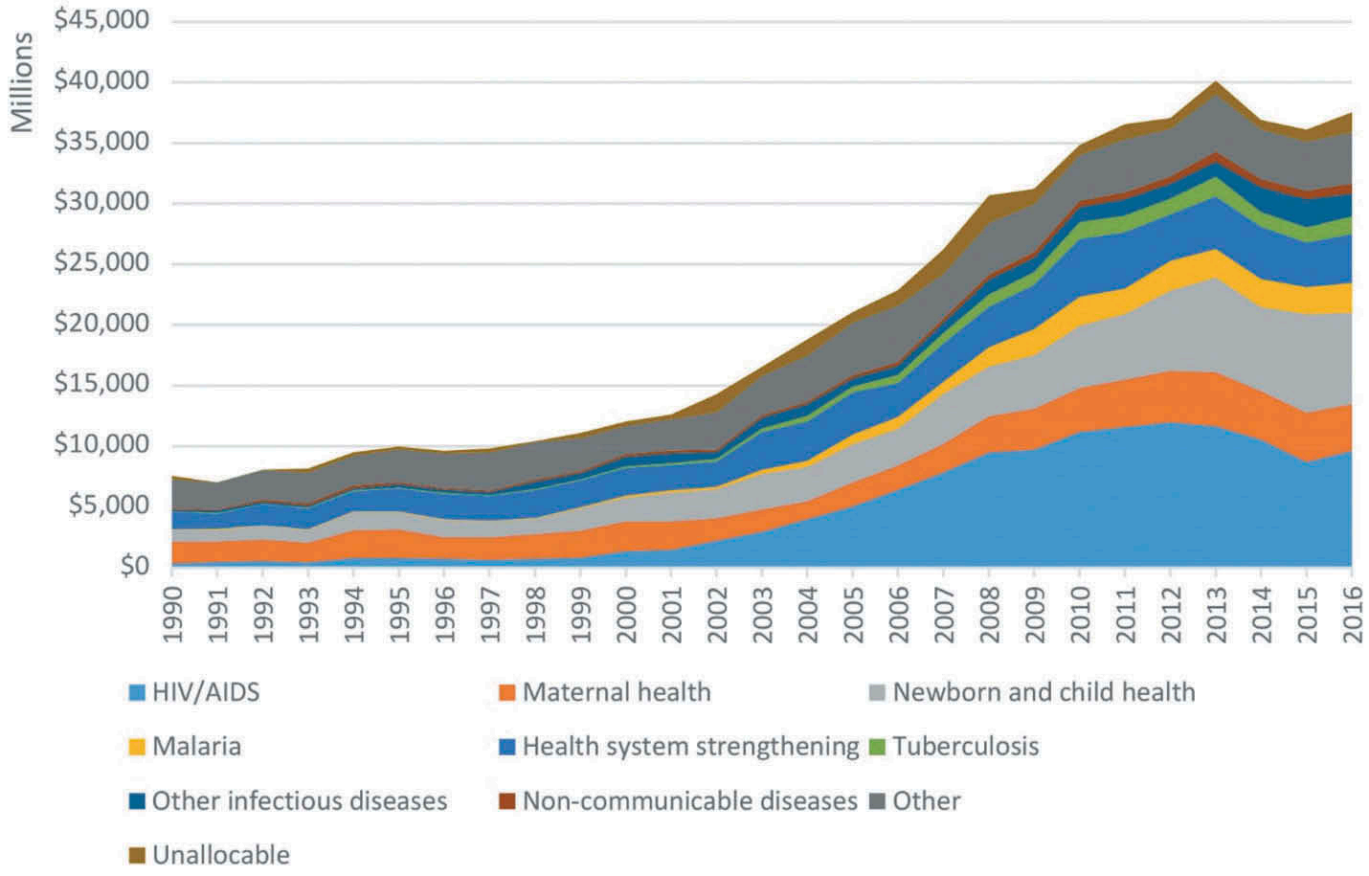
Development assistance for health, distributed by focus area (IHME, 2018).

Development assistance for health (DAH) has increased significantly since 1990. However, as can be seen in Figure 1 the large majority of additional funding has gone to the fight against specific diseases or themes such as HIV/AIDS [1]. Furthermore, interventions have often been set-up with little involvement of the state, which led to the creation of so-called parallel systems. Consequently, little money and attention has been dedicated to strengthening the public health systems in developing countries. The lack of a well-functioning public health system can have devastating consequences, as was brutally demonstrated during the 2014–2016 Ebola outbreak in West Africa [2,3].

The importance of health system strengthening (HSS) has already been acknowledged for decades, with an important milestone being the *Alma Ata Declaration* from 1978. This declaration aimed to reach ‘health for all’ by 2000 and stressed the importance of primary healthcare and HSS to reach this goal [4]. However, the principles of Alma Ata soon lost prominence and donors have provided much more attention and funding to fight specific diseases rather than to HSS. This trend was reinforced by an unprecedented growth of so-called Global Health Initiatives in the beginning of the new millennium, the most important ones being the Global Fund to fight HIV/AIDS, Tuberculosis and Malaria (hereafter the Global Fund), the Global Vaccines Initiative (GAVI) and the US President’s Emergency Plan for AIDS Relief (PEPFAR). These initiatives have massively increased the resources for global health, but have been using rather disease-specific, parallel approaches. Yet, since the mid-2000s, academics and policymakers have increasingly stressed the limitations of these approaches, which led to a renewed consensus on the need for HSS.

However, despite the increased attention for HSS, there is no common understanding on what the term exactly entails [5,6]. Consequently, donors have different interpretations and use different strategies to implement it. Moreover, the discursive importance attached to HSS in policy documents does not necessarily translate into increased support for HSS at country-level. Several authors claim that HSS is often interpreted and implemented in a very narrow, instrumental way, using well-targeted and specific interventions with clear, measurable outcomes [7–9]. This is in strong contrast to a broader conceptualization of HSS focused on social, societal and political dimensions.

The European Union (EU) and its Member States have been important donors, providing about a quarter of all DAH [1]. Within their policy documents, European donors tend to focus a lot on strengthening health systems. The Council conclusions of 2010 on the EU role in Global health even explicitly stated that *‘the Council calls on the EU and its Member States to act together in all relevant internal and external policies and actions by prioritizing their support on strengthening comprehensive health systems in partner countries’* [10].

Nevertheless, little is known about how this European support for HSS in policy documents translates into concrete policies at partner country-level. Furthermore, as development cooperation is a shared policy between the EU and its 28 Member States, it remains unclear to what extent European donors share a similar approach on this matter. The limited available research on European development cooperation seems to stress the differences between the individual European donors. An often made distinction is the one between the more progressive Nordic and like-minded countries and the more traditional Southern Member States [11,12]. Nevertheless, European approaches have not been systematically and comparatively analysed, neither in general nor with a specific focus on the sector level.

Another related theme is the level of ‘distinctiveness’ or ’uniqueness’ of European donors. This relates to the question to what extent there is – despite the differences among them – at least some degree of similarity among European donors, making them different from other donors. Certainly, there are differences with the emerging donors, which have challenged the ‘Western’ consensus on international aid when it comes to issues such as political conditionality [13,14]. In addition, European donors are said to be inspired by normative principles and they have paid more attention to the aid effectiveness principles than is the case for the US [15–17]. In health, in particular, some authors referred to an Atlantic fault-line in thinking on health systems, which is linked to the competing public health ideologies across the Atlantic ocean [9,18]. The US is generally considered to be supportive of technology-oriented disease-specific solutions, providing funding through parallel systems and NGOs. European donors on the other hand are considered to promote coordinated public sector aid models, favouring sector-wide approaches and budget support models. Yet, Storeng [9] claimed that the transatlantic distinction is eroding in favour of a more narrow, technology-focused and market-based interpretation of HSS.

Building on these insights from the field of international health assistance as well as EU-Studies, the main goal of the PhD research was to get an in-depth understanding of the approaches on international health assistance (IHA) of several European donors, with a specific focus on the partner country-level.

The main research question of my dissertation was: **‘What is the European approach to international health assistance?’** In order to answer this question, the following sub-questions were formulated:

1. **What are the approaches of European donors towards international health assistance?**
2. **To what extent are there similarities and differences between European donors in this regard?**

## Empirical embarkation

It would have been unfeasible to research all European donors’ health assistance approaches towards the whole developing world through all channels and at all times. Consequently, I had to narrow down the focus. I analysed and compared the IHA approaches of 13 European donors in six empirical settings (Table 1). It concerns EU itself, 10 EU Member States (France, the UK, Belgium, the Netherlands, Sweden, Denmark, Ireland, Italy, Spain and Germany), ^1^1As the referendum on the Brexit took place in the middle of the research and as it is not clear yet what this will imply for the development cooperation policies of the UK and the EU, the UK is considered an EU Member State as any other in this dissertation. Flanders (a federated entity of Belgium) and Switzerland.^2^2While Switzerland is not part of the EU, it was integrated in the analysis because it shares a similar history with the EU donors in the health sector of Mozambique and because existing research suggested that Swiss development cooperation policy is relatively ‘Europeanized’ [36]. These are the European donors that have financed the most DAH over the past years. However, not all Member States’ approaches were discussed in every empirical setting.

**Table 1.** Overview of empirical settings.

		EU	FR	UK	BE	FL	NL	SE	DK	IE	IT	ES	DE	CH
Headquarters		x	x	x	x	x	x	x	x	x	x	x	x	x
Bilateral cooperation in partner countries	DRC	x	x	x	x			x						
	Ethiopia	x		x			x			x	x	x		
	Mozambique			x		x	x		x	x	x			x
	Uganda			x	x		x	x		x	x			
The Global Fund		x	x	x	x				x				x	

The first setting concerns the European donors’ approaches at headquarters level. Relevant documents and aid figures at headquarters level were discussed to illustrate European donors’ vagueness and ambiguity on HSS. While certainly important, this context was not the main focus of the PhD as it served as a broader context for the other two, more specific empirical contexts.

The second group of empirical settings was the core of the PhD and concerned the bilateral cooperation of European donors in four partner countries in Sub-Saharan Africa. I focused on Sub-Saharan Africa as this continent suffers the most from health problems and receives most DAH from (European) donors [1]. Specifically, I examined European donors’ approaches in the DRC, Ethiopia, Uganda and Mozambique. These countries were selected because at least five European donors assigned health as a priority sector of their bilateral aid in these countries. Consequently, by focusing on these four countries, a maximum amount of European donors’ approaches could be analysed. First, it could be analysed how different European donors were reacting in the same country. Second, it could be researched how a certain donor was behaving in different partner countries and to what extent some general trends about this donor’s approach can be observed across these countries.

The last empirical setting concerned the multilateral cooperation via the Global Fund. The EU and several of its Member States have played an important role in the development of the Global Fund and have been contributing considerable amounts of money to it. However, this seems to contradict with the European pleas for HSS as the Global Fund has a disease-specific approach and its efforts on HSS have been limited. During interviews in the partner countries, several respondents referred to this ambiguous relation between European donors and the Global Fund. Consequently, I dedicated a separate article to it [19] which was also included in the PhD dissertation.

## Methods

The research for my dissertation was conducted through an abductive research approach. Abduction reasons at an intermediate level between deduction and induction [20]. Instead of following a linear ‘first this, then that’ logic, it implies a more cyclical research process during which literature review, data generation, data analysis and research design mutually influence each other [21]. Abduction stems from the pragmatist research tradition [22] and allowed me to conduct problem-driven and practice-oriented research and to take a holistic perspective on the European involvement in international health assistance.

Empirical data were generated and analysed through a mixture of research methods. A first method was semi-structured expert interviewing. In total, interviews were conducted with 123 respondents. While some explorative interviews were conducted at headquarters level (in 2014–2015), most of the interviews were conducted during four fieldwork trips in November–December 2015 (the DRC and Ethiopia) and March–April 2017 (Uganda and Mozambique), as well as additional Skype interviews in early 2017 with respondents in the DRC. The group of respondents included representatives from European donor agencies, non-European donor agencies, the Ministries of Health, civil society organizations and some local academic researchers. This broad range of respondents allowed me to get both internal and external perspectives on European IHA approaches. In May–June 2017, additional interviews were conducted over Skype to obtain more specific information on the relations between EU donors and the Global Fund. All interviews were recorded on audiotaped, fully transcribed and analysed by using NVivo software.

In addition to the interviews, policy documents proved to be another important source of data. A variety of documents was consulted, which focused on the headquarters level (e.g. development cooperation or global health strategies) or on the specific partner countries (e.g. donors’ country-specific development cooperation strategies, program documents or joint evaluation documents). Most of these documents were publicly available on donors’ websites and other – more specific – documents were provided by interviewees. The documents were analysed through a close and iterative reading. To complement the data from interviews and policy documents, I also conducted some descriptive statistical analysis on European donors’ DAH, based on the ‘Development Assistance for Health Database 1990–2017’ from the IHME [1].

## Analytical framework

To analyse the European approaches in four partner countries, an original analytical framework was developed. This framework built on the often-made distinction between so-called ‘vertical’ and ‘horizontal’ approaches. The vertical approach implies that funding and attention are mainly going to disease-specific interventions, which often leads to quick, visible and measurable results. The horizontal approach entails a focus on strengthening basic health care and the wider health system. This is claimed to be more sustainable in the long term, but it is also more abstract, as the results are difficult to measure. The past decades have shown a continuous debate on the advantages and disadvantages of both approaches, e.g. [23–28].

**Figure 2.**
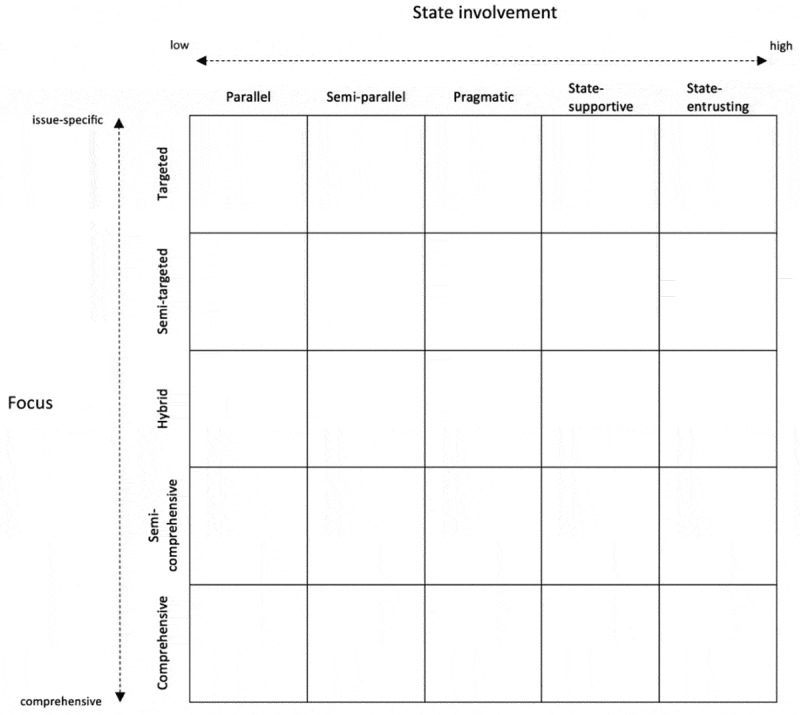
Analytical framework.

However, while often used, the vertical–horizontal dichotomy is somewhat problematic. First, the terms are ambiguous, as they refer to a wide range of phenomena [29,30]. Second, by framing the debate as a dichotomy, one creates the impression that an IHA approach is either ‘vertical’ or ‘horizontal’, while hybrid approaches are also possible. To allow for a more fine-grained analysis of the different European approaches, I, therefore, developed a framework which exists of two continuums (Figure 2).

The first continuum concerns the focus. This links with the more conventional interpretations of the vertical versus horizontal debate as being specific, narrow and non-integrated versus comprehensive and integrated. Given the problematic use of these terms, I prefer to use the terms ‘issue-specific’ and ‘comprehensive’, which are the two ends of the top-down continuum of the framework. The continuum is divided into five separate categories:

**Targeted**: Particular focus on a specific disease or health problem

**Semi-targeted**: Focusing on a particular disease or health problem, but taking into account the wider system

**Hybrid**: Balancing a focus on specific diseases or health problems with a wider focus on the health system

**Semi-comprehensive**: Focusing on the overall health system, while still prioritizing certain diseases or health problems

**Comprehensive**: Holistic focus on the overall health system

The second continuum, state involvement, links to the deeply political question to what extent donors support the state and the existing country systems. Although the vertical-horizontal distinction mostly concerns the focus of IHA, some authors also use it to denote the extent to which donors make use of existing structures [24]. In addition, *‘the relative power states and private sector actors should have over the stewardship of health systems’* is also considered to be one of the major fault lines in the debate on HSS [6]. The dimension on state involvement thus refers to the extent to which donors support governmental policies (e.g. national health strategies), existing state structures (e.g. Ministry of Health, national procurement systems), or even official policymakers (e.g. members of government). These dimensions of state involvement can be conceptualized at both central and local levels. The state involvement is the left-right continuum of the framework and is divided into the following five categories:

**Parallel**: Working through parallel systems without involving the state

**Semi-parallel**: Working through parallel systems while involving the state to a limited extent

**Pragmatic**: Involving the state to the extent possible and consulting the governmental institutions as ‘one of the partners’

**State-supportive**: Using the existing system to a large extent and supporting the governmental institutions in developing and/or implementing its plans

**State-entrusting**: Entirely supporting the state

While it may seem plausible that both dimensions correlate, it is important to analytically distinguish between them. First, this two-dimensional approach allows us to clarify the current conceptual confusion around a donor’s IHA approach and to make a more precise assessment of it. Second, one could imagine that, at least theoretically, donors have opposite approaches on both dimensions. On the one hand, a donor could have an issue-specific *focus* while at the same time closely involving the *state* through collaborating with the government and the existing public health system. This would for example be the case if a donor supports the third country government to implement its specific HIV/AIDS policy. On the other hand, a donor could theoretically have a relatively comprehensive *focus* which is nevertheless implemented entirely parallel without involving the *state*. This would for instance be the case if a donor is supporting NGOs to provide primary healthcare services, without involving the government officials and without taking into account the existing policies and structures.

The combination of these two continuums results in 25 different possibilities categories. Being ideal types, all these 25 categories are not necessarily expected to occur in reality. Especially the categories at the extremities are thought to be less common in reality, as donors generally consider them to be either undesirable (entirely targeted or parallel) or unrealistic (entirely comprehensive or state-entrusting). The European approaches will thus mainly vary between the middle nine approaches.

## Results and discussion

### Overview of the empirical settings

**Figure 3.**
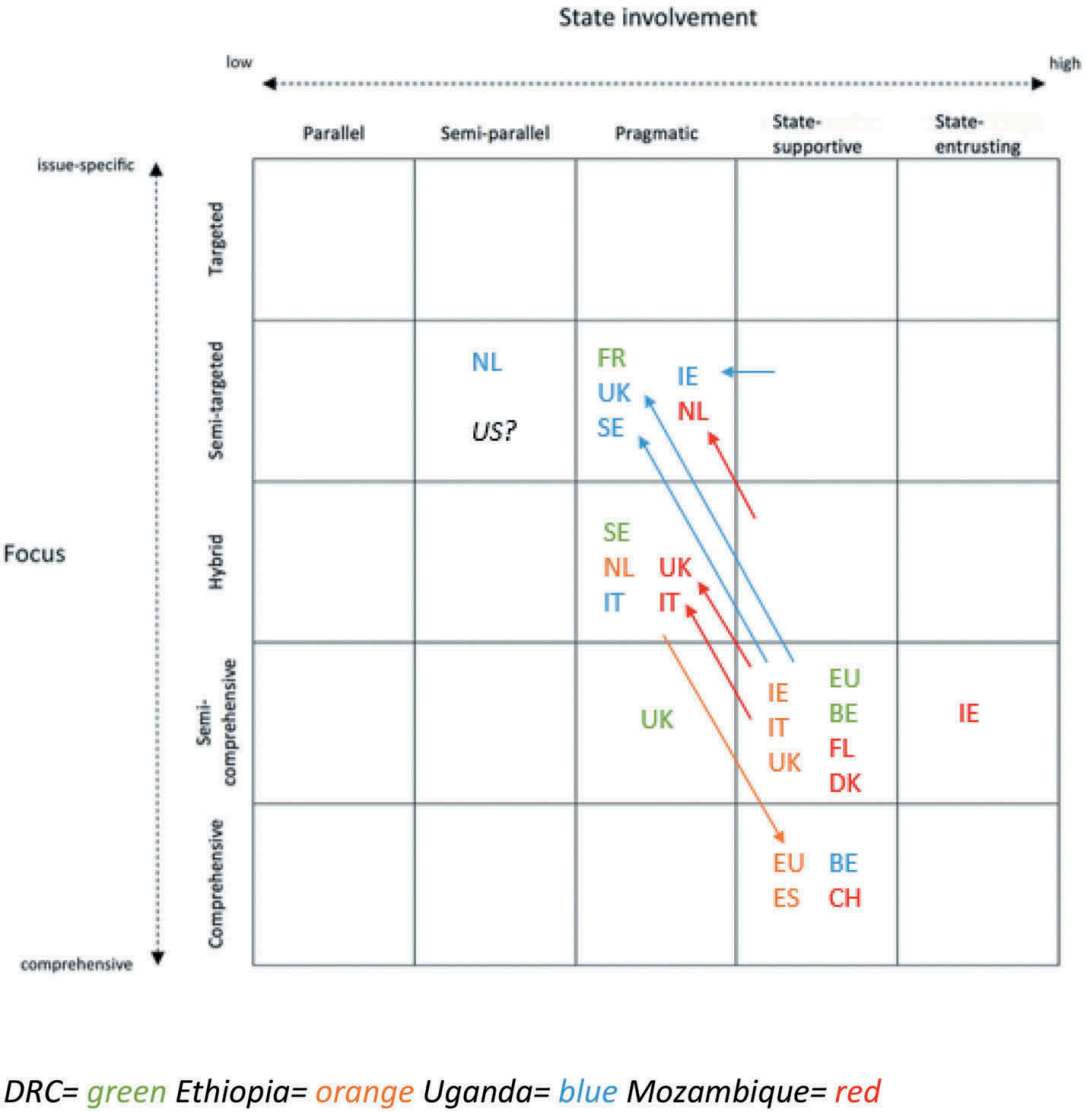
Summary framework.

The analytical framework was applied to European donors’ health assistance in the DRC, Ethiopia, Uganda and Mozambique. Figure 3 provides a summary of how the analytical framework was applied on European donors’ approaches in the four countries. The contexts differed quite substantially, which also impacted on donors’ approaches. In the next paragraphs, I will provide a short summary on the approaches in each setting.

Due to the fragile context of the **DRC, high levels of corruption** and the low level of trust in the Congolese government and structures, the existence of and discussion on different approaches was the most prominently present in this country. In principle all donors supported the idea of HSS, but there seemed to be different interpretations of it. While some donors wanted to collaborate with the state institutions and work as much as possible through the existing structure, others were more reluctant and thought that the system had to be built first before it could be used. These different interpretations featured prominently in the debate on the procurement and distribution of medicines, with some donors using the existing national system as much as possible and others refusing to make use of it until it proved to be working more efficiently. Overall, the approaches of European donors varied, with Belgium and the EU having a state-supportive approach, and the UK, Sweden and France having a more pragmatic approach. Also in terms of focus, approaches ranged between semi-targeted and semi-comprehensive.

In **Ethiopia**, there was a relatively good relation of trust between donors and the Federal Ministry of Health. This was mainly due to the strong ownership and leadership of the FMOH, which succeeded in managing the ‘aid jungle’ by aligning the donors with its own plans and priorities. All European donors were contributing to the pooled MDG/SDG performance fund. Consequently, most European donors had a state-supportive approach. Differences in focus related to specific objectives donors want to obtain, which can be manifested by pushing for certain topics in discussions about the MDG/SDG performance fund, but also by funding additional-specific projects/programmes through which a specific theme is stressed. For example, the Netherlands focused a lot on sexual and reproductive health and rights and transferred a relatively big amount through NGOs, in addition to the MDG/SDG performance fund (which also resulted in a more pragmatic approach than other donors).

In **Uganda** European donors used to be the strongest supporters of the Sector Wide approach (SWAp) and Sector Budget Support (SBS) in the late nineties and the beginning of the 2000s, which implied a relatively comprehensive focus and a high level of state involvement. However, over the years the relation between SWAp supporters and the government started deteriorating, due to a de-prioritization of health by the government and increasing concerns of corruption. At the same time, European domestic governments started to focus increasingly on value-for-money and quick results, which led to less commitment to the principles of the SWAp and SBS. When a big corruption scandal at the Office of the Prime Minister happened, all donors ended their direct support to the government. Consequently, the SBS in the health sector was also suspended, which made some donors change their approach. Belgium was the only donor that still applied a comprehensive and state-supportive approach. Despite the fact that it also suspended the SBS, it has implemented programmes which aim to improve the entire health system and which are implemented in close collaboration with the Ministry of Health. The other European donors had a more issue-specific focus with a lower level of state involvement.

In **Mozambique**, most European donors used to contribute to the pooled fund PROSAUDE. However, over the years PROSAUDE encountered several challenges which led to severe discussions on its reform. However, this reform process was hampered by other factors. Due to a big debt scandal in 2016, donors completely lost their trust in the government systems, which made most donors decide to suspend all aid that was provided directly to the government, including the funding for PROSAUDE. In addition, the Global Financing Facility (GFF) became an additional important initiative in the health sector of Mozambique and was regarded by some as an attractive alternative for PROSAUDE, given its more specific focus on women and child’s health and its attention for cost-effectiveness and results. The ongoing discussions on the reform of PROSAUDE and the launch of the GFF made clear that different positions existed among European donors. While most donors (Ireland, Switzerland, Denmark, Flanders and Italy) continued their support for PROSAUDE, the UK and the Netherlands decided to stop providing funding, which implied more issue-specific and pragmatic approaches. On top of that, there were also clear differences in terms of the relative importance attached to PROSAUDE, as well as differences in focus and level of state involvement in the complementary programmes.

As mentioned earlier, I also investigated the relation between European donors and the **Global Fund**, with a focus on the debate on HSS. This research started from the puzzling observations during fieldwork, where interviewees at European agencies complained about the negative effects of the Global Fund on countries’ health systems. As European donors themselves contribute significantly to the Global Fund, I wanted to better understand this relation. The findings indicated a ‘love–hate relationship’. European donors have loved the Global Fund’s innovative institutional set-up and its ‘saving lives’ approach involving quick results. However, over the years they have become more critical about the Global Fund’s narrow focus. Consequently, they have increasingly advocated a shift towards more HSS. This has been partly successful at the headquarters level, most notably the incorporation of concrete HSS commitments in the Global Fund’s strategic documents, but challenges at local level constrain the translation into funding and implementation measures. The key tension is that ‘horizontalizing’ the Fund remains challenging, because the ‘specificities’ that make the Global Fund so successful and attractive, are precisely those that also impede moves towards HSS.

As a detailed analysis of the European approaches in each empirical setting can be found in the separate chapters of the dissertation, I will focus in the remaining part of this PhD review on the overall findings.

### Heterogeneity among european donors

A first important finding of the PhD research was the heterogeneity among European approaches. As can be seen in Figure 3, individual donors’ approaches can differ geographically and temporally. Differences between partner countries can be seen from the different colours in Figure 3, which represent the four partner countries. While several European donors were present in the health sector in two or more partner countries, this did not mean that they had exactly the same approach in all settings. For example, the UK was present in four partner countries and its approach has been classified differently in each country. Furthermore, differences could also be observed over time, as can be seen from the arrows on the summarizing framework. While the approaches in the DRC and Ethiopia remained fairly stable, there has been a change in approach among some of the donors in Uganda and Mozambique over the past years. For example, the Dutch approach in Mozambique changed from hybrid and state-supportive to semi-targeted and pragmatic.

However, despite these geographic and temporal differences, certain patterns could be identified in the approaches of each individual donor. Some donors stay within the same ‘region’ of the framework and do not move much. As such, their position in the framework remains relatively stable. For example, Belgium is typically situated at the bottom right of the framework. Some donors display a certain pattern in how they move within the framework. For instance, a number of countries have typically moved towards the upper left part of the framework. Consequently, I distinguished four ‘types’ of European donors.

Belgium, the EU, Switzerland, Spain, Denmark and Flanders are classified as type 1, which can be referred to as ‘hardline health system strengtheners’. These donors were classified as either semi-comprehensive and state-supportive or comprehensive and state-supportive. The donors within this group seem to apply this approach regardless of the governance situation. This implies that even in a challenging governance situation, they endeavour to have a comprehensive focus and to have a rather high level of state involvement.

The second type of donors is the Netherlands, Sweden and the UK. Their approaches are in general more targeted and less state-supportive than the former type of donors. Furthermore, their approaches have also changed over time in Uganda and Mozambique. Consequently, they can be considered to be ‘flexible’ in the sense that the focus and level of state involvement is likely to change in light of the governance situation of the partner country. When there is a favourable context, this type of donors contributes to pooled funds or sector budget support, which implies a relatively comprehensive and state-supportive approach. However, in case the context is (or becomes) less favourable, these donors work through other partners such as NGOs and UN agencies that are often implementing more targeted programs. In the latter case, there is still a pragmatic level of state involvement, but it seems to be more important that results can be made on specific focus areas.

Ireland and Italy can be considered a third type of donors. In general, these donors tend to be more state-supportive than the flexible group but they have a less profound approach on it than the hardliners. Furthermore, the group cannot be characterized by a stable focus, as donors are sometimes more issue-specific (cf. flexible group) and sometimes more comprehensive (cf. hardliners).

France does not belong to any of the former types, as it is has a very particular stance. France is an important donor to the Global Fund and GAVI and has decided to complement its financial presence with an active presence in the policy dialogue in these organizations, both at the level of the board and at the level of partner countries. Within this policy dialogue, France seems to be advocating for a more comprehensive focus and a higher level of state involvement. While the French approach was only investigated in the DRC, the empirical data on the engagement of France in the Global Fund as well as policy documents at headquarters level and existing (grey) literature proved to be relevant additional data to substantiate this finding.

### European ‘unity’ in diversity?

The above-mentioned classification of European donors shows that there is quite some heterogeneity among European donors. Nevertheless, despite these differences at the specific level, the research also revealed that there is still a certain degree of ‘unity’ among European donors at a more general level, which contrasts with other donors such as the US. Consequently, while nuancing the existence of a ‘European’ approach, this research also confirms the transatlantic divide discussed in literature.

The relative unity among European donors and its distinctiveness in relation to the US were prominently discussed in the research on the Global Fund. The article clearly showed the varying approaches on HSS between the European donors and the US. Already in the initial stages of the Global Fund’s development, there seemed to be different opinions on the matter. But since the Global Fund was thought to provide ‘complementary’ support on top of bilateral funding, this was not considered to be problematic at that time. However, over the years, the Global Fund became a more powerful organization and its targeted and parallel approach increasingly got criticized. As the Global Fund appeared to have negative effects on countries’ health systems which European donors themselves have tried to build and strengthen, European donors increasingly tried to ‘horizontalize’ the Global Fund. The US on the other hand has never been a strong advocate of broadening the approach of the Global Fund.

The contrast between European approaches and the US was also evidenced in the analysis of approaches in the partner countries. Regardless of the capacity or leadership of the state, European donors in general were having a rather comprehensive focus and a relatively high level of state involvement. In Ethiopia, Uganda and Mozambique, European donors were the earliest and most generous supporters of, respectively, the MDG/SDG PF, the health SBS and PROSAUDE. In the fragile and challenging context of the DRC, European donors acknowledged the importance of making use of the system as much as possible, which was manifested most clearly in the debate on the procurement and distribution of medicines. On the other hand, the US did not participate in any of the pooled funding arrangements in Ethiopia, Uganda and Mozambique. And in the DRC, the US shared the opinion with the Global Fund and GAVI that the national system for procurement and distribution of medicines had to be built first before it can be used. Consequently, the general approach of the US can be considered to be semi-targeted and semi-parallel. Compared to the US, all European donors’ approaches are thus located more towards the right and bottom side of the framework.

However, this transatlantic divide also needs to be nuanced as some degree of ‘convergence’ can be noticed between the approaches at both sides of the Atlantic Ocean. On the one hand, European donors seem to have become less profound supporters of HSS in recent years. As clarified earlier that some European donors are more ‘flexible’ and have moved away from HSS oriented approaches. Importantly, even within the so-called ‘hardliners’, there are indications that HSS is increasingly contested (e.g. in Belgium). The research on the Global Fund also suggested that HSS may perhaps not be as important a political priority for European donors as often thought/pretended, as they continue supporting the organization even though it remains questionable whether the proclaimed efforts for more HSS can be successful. On the other hand, the US seemed to have opened up its approach a bit towards HSS. In all of the partner countries, respondents of the US mentioned that there has been a gradual shift towards a more comprehensive focus and a higher level of state involvement over the years. At the same time, the recent, progressive move of the Global Fund towards more HSS at headquarters also implies that the US (which is a very important donor of the Global Fund) has also agreed upon a (limited) broadening of the approach.

### Towards an understanding of the findings

This dissertation provided an in depth *analysis* of European donors’ approaches and a full *explanation* went beyond the scope of the research. Nevertheless, I can touch upon three majors factors that help to better understand the (evolving) approaches, differences and similarities between European donors and open avenues for further research.

First, the research clearly demonstrated the importance of partner country-related factors. In particular, respondents often referred to the governance situation in the partner country when discussing (certain changes in) European donors’ approaches. The DRC and Ethiopia could be considered to be two ‘extremes’ when it comes to the governance situation. In the DRC, the fragile context, the high level of corruption and the low level of trust in the Congolese government and structures made that none of the donors wanted to contribute to pooled funding arrangements. In Ethiopia on the other hand, the leadership of the Ministry of Health made that a relatively good relation of trust existed and that European donors have been contributing to the pooled SDG performance fund. The governance situation of Mozambique and Uganda can be considered to be somewhere between these two extremes. In both countries, there used to be a relatively high level of trust between donors and the government. However, over the years, the governance situation deteriorated. In a context in which donors have already been struggling for years, the occurrence of certain corruption scandals became the decisive moment for donors to change their approaches.

Second, the research showed the importance of changes in international thinking on development assistance the way European donors have reacted to these. In the 1990s and early 2000s, the international development community paid increased attention to ownership, harmonization and alignment, which was manifested through the development of the SWAps and the contributions to pooled funds and (sector) budget support. European donors attached great importance to the Paris Declaration and soon became frontrunners in the aid effectiveness agenda [31]. This has also facilitated a certain level of unity among European donors’ approaches in IHA. As was discussed in the dissertation, European donors were the earliest supporters of the pooled funds and SBS in Ethiopia, Uganda and Mozambique. By the end of the 2000s, however, the international support for ownership, alignment and harmonization started to wane and increasingly got ousted by a strong focus on value-for-money and quick results that can directly be attributed to individual donor activities and which can be easily communicated to the public [32]. This tendency has very clearly manifested in the health sector, where practices are being measured and evaluated using quantitative indicators such as the amount of bed nets provided, the amount of people vaccinated, and – ultimately – ‘the amount of lives saved’ [33,34]. As shown in this research, European donors have not been immune to this international tendency to prioritize value-for-money and quick results. Especially in Uganda and Mozambique, donors seemed to have lost faith in the principles of ownership, harmonization and alignment, given the waning support for the Ugandan SWAp and SBS, and the Mozambican PROSAUDE. Several respondents were referring to the fact that their headquarters increasingly focused on results and accountability, which has pushed the principles of ownership, harmonization and alignment more to the background. Nevertheless, this tendency to focus less on the latter principles and more on value-for-money and quick results appeared to be more apparent for certain European donors than for others.

While the two above-mentioned factors certainly help to understand why the preference for HSS among European donors is rather fragile and ambiguous, they fail to explain the particular differences between European donors. Consequently, I suggest that one also needs to investigate the domestic factors of European donors that differ between them and can explain the different approaches on HSS. These factors can include (changes in) the composition of national governments, the (lack of) room of manoeuvre for local delegations, the institutional organization of development policy (and of social and health policy), the power of national parliaments and civil society organizations, economic and foreign policy interests, etc.. My research already revealed domestic factors in Belgium, including the collaboration between and like-mindedness among several Belgian actors, which might explain the Belgian position as being a hardline health system strengthener [35]. Yet, the investigation of the domestic factors for all European donors went beyond the scope of this research and will need to be subject to further research.

## Conclusion

The main goal of this dissertation was to get an in-depth understanding of the IHA approaches of European donors. The systematic, fine-grained analysis showed that there is significant heterogeneity of European approaches on international health assistance. Donors’ approaches differ between partner countries and they can change over time. Yet, certain patterns can be identified that characterize individual donors. Consequently, four ‘types’ of European donors were distinguished, which vary in their level of support to and involvement of recipient states and in their level of focus on specific diseases or health issues. Despite this high level of heterogeneity at a specific level, the PhD research nevertheless also showed that there is still a general degree of ‘unity’ among European donors. These distinctive similarities become even more evident when comparing European donors with the US. Yet, there are signs that the ‘transatlantic’ divide on the issue of health system strengthening may be converging, as several European donors are increasingly focusing on cost-effectiveness and direct results, which is traditionally more associated with the US and global health initiatives.

In sum, this dissertation illustrated the heterogeneity of European approaches and the tendency to move away from state-supportive, comprehensive HSS investments. Consequently, the EU as a whole seems to play a limited role in bringing health system strengthening more to the forefront in international health assistance. If European donors still want to ‘walk their talk’ on HSS, they will have to reconsider the way they are providing health assistance in fragile governance contexts and find innovative ways to strengthen the system. In addition, they have to become more involved in the follow-up of multilateral organizations both at headquarters and local level.

This research has been one of the first studies that bridged insights from the field of EU-studies with the field of international health assistance, by thoroughly investigating and comparing the European IHA approaches. Throughout the empirical research, I also made an important analytical contribution, as I developed a new framework to analyse donors’ IHA approaches, which consists of two continuums being the *focus* of IHA and the *state involvement*. The application of the framework confirmed the relevance of the two separate continuums. The fact that not all the donors’ approaches were classified in the diagonal from the upper left corner to the bottom right corner illustrate that there is no one-to-one relationship. Nevertheless, the application of the framework also showed that in most cases, the position of donors on both continuums seemed relatively closely related to each other. A high level of state involvement often involved a rather comprehensive focus, as is the case when donors are contributing to pooled funds or SBS. When donors have a rather pragmatic level of state involvement, for example, when they are channelling funding through UN agencies, this often also implied a more targeted approach. This framework can be further developed by focusing especially on the relation between the two continuums.

The broad perspective on several European donors and several empirical settings have had the downside that not everything could be investigated in depth. Furthermore, the focus of the research was mainly on the donor side and less on the domestic factors in the partner countries. Consequently, future research could focus more in depth on one or a few European donors, with more attention for the domestic politics and institutions which might explain the preference for a certain approach. For example, a closer look at the UK could be all the more relevant in the light of the Brexit. Also, the role of post-colonial influences on donors’ DAH could be investigated more in depth. In addition, ethnographic research could focus more on the partner country itself and investigate how the political, cultural, economic, geographical and societal factors are influencing the health system and the health assistance.

## Paper context

While European donors are considered to be strong supporters of health system strengthening (HSS), little is known about how this support translates into concrete policies at partner country level. This research illustrated the heterogeneity of European approaches and the tendency to move away from state-supportive, comprehensive HSS investments. If European donors want to walk the talk on HSS, they will have to find innovative ways to strengthen the health system in fragile governance contexts.
